# Genomic identification and evolutionary analysis of chemosensory receptor gene families in two *Phthorimaea* pest species: insights into chemical ecology and host adaptation

**DOI:** 10.1186/s12864-024-10428-6

**Published:** 2024-05-18

**Authors:** Ruipeng Chen, Junjie Yan, Jacob D. Wickham, Yulin Gao

**Affiliations:** 1 State Key Laboratory for Biology of Plant Diseases and Insect Pests, Institute of Plant Protection, Chinese Academy of Agricultural Sciences, Beijing, 100193 China; 2 A.N. Severtsov Institute of Ecology and Evolution, Russian Academy of Sciences, Moscow, 119071 Russia; 3 Department of Entomology, Rutgers University, 93 Lipman Drive, New Brunswick, New Jersey USA

**Keywords:** *Phthorimaea operculella*, *Phthorimaea absoluta*, Chemosensory receptors, Transcriptome analysis, Gene expression profiles

## Abstract

**Background:**

Insects rely on sophisticated sensitive chemosensory systems to sense their complex chemical environment. This sensory process involves a combination of odorant receptors (ORs), gustatory receptors (GRs) and ionotropic receptors (IRs) in the chemosensory system. This study focused on the identification and characterization of these three types of chemosensory receptor genes in two closely related *Phthorimaea* pest species, *Phthorimaea operculella* (potato tuber moth) and *Phthorimaea absoluta* (tomato leaf miner).

**Results:**

Based on manual annotation of the genome, we identified a total of 349 chemoreceptor genes from the genome of *P. operculella*, including 93 *OR*, 206 *GR* and 50 *IR* genes, while for *P. absoluta,* we identified 72 *OR*, 122 *GR* and 46 *IR* genes. Through phylogenetic analysis, we observed minimal differences in the number and types of ORs and IRs between the potato tuber moth and tomato leaf miner. In addition, we found that compared with those of tomato leaf miners, the gustatory receptor branch of *P. operculella* has undergone a large expansion, which may be related to *P. absoluta* having a narrower host range than *P. operculella*. Through analysis of differentially expressed genes (DEGs) of male and female antennae, we uncovered 45 DEGs (including 32ORs, 9 GRs, and 4 IRs).

**Conclusions:**

Our research provides a foundation for exploring the chemical ecology of these two pests and offers new insights into the dietary differentiation of lepidopteran insects, while simultaneously providing molecular targets for developing environmentally friendly pest control methods based on insect chemoreception.

**Supplementary Information:**

The online version contains supplementary material available at 10.1186/s12864-024-10428-6.

## Introduction

Chemical sensing in insects relies on the participation of chemoreceptor organs, which are divided into two categories: olfactory receptors and gustatory receptors [1]. The process of insect olfaction recognition is complex and involves the participation of multiple chemical sensory genes. This process occurs at the level of the peripheral olfactory nervous system and involves chemosensory genes such as odorant receptors (ORs), ionotropic receptors (IRs), and gustatory receptors (GRs), along with other chemical sensory receptor proteins that recognize odorant molecules and deliver them to receptors [2]. The main organ involved in olfaction is the antennae, while the gustatory organs include the maxillary and labial palps, proboscis [3], legs [4], and ovipositors [5]. In addition, some insects also have gustatory receptor neurons distributed on the wing margins that can function as gustatory receptors to perceive taste [6]. Many substances, such as acids, carbon dioxide or carbonic acid, and even water, can be perceived through both taste (gustation) and smell (olfaction) [7]. Due to technical limitations, there is currently more research on the process of insect olfaction than on the signal transduction pathways of gustatory receptors [1].

The length of insect ORs is about 400–450 amino acids, consisting of 7 transmembrane domains [8]. Insect odorant receptors are structurally very different from mammalian odorant receptors, which belong to the G protein-coupled receptor (GPCR) gene family and have the typical characteristics of 7 transmembrane helical domains [9]. However, insect odorant receptors have opposite topologies, with their N-terminus located inside the cell and their C-terminus located outside the cell membrane [10]. Gustatory receptors (GRs) primarily function in taste perception. Insects use GRs to assess the quality of food and their environment, as well as to avoid toxic and harmful chemicals. Some of these GRs are crucial for insects to sense CO_2_. Taste receptors are expressed on neurons of taste organs and recognize non-volatile chemicals [11]. The first GRs in insects were identified in the genome of *D. melanogaster* [2]. With the completion of genome sequencing for various insects, research on taste receptors has revealed that the secondary structure of taste receptors is similar to that of odorant receptors, with multiple transmembrane domains and the same membrane topology as ORs [12]. According to the nature of ligands, insect taste receptors can be classified into bitter receptors, carbon dioxide (CO_2_) receptors, and sugar receptors. Benton et al. identified the first ionotropic receptor gene family in insects from the genome of fruit flies [13]. Current studies on the function of ionotropic receptor genes in *Drosophila melanogaster* suggest that IRs are only expressed on olfactory sensory neurons (OSNs) in the olfactory cone sensilla and are involved in the detection of various amine and acid compounds. In addition to olfactory perception, IRs in fruit flies also participate in taste recognition, primarily perceiving compounds such as amino acids, amines, salts, and acids, which can indicate the nutritional value and quality of food to varying degrees. Gustatory receptor neurons (GRNs) responsible for taste perception exist in various taste organs, including the labellum, pharynx, legs, wing margins, and abdomen of fruit flies. IRs have been identified in all confirmed taste organs [14]. Furthermore, some studies suggest that IRs may also be involved in the perception of temperature and humidity in the environment [15–17].

The potato tuber moth, *Phthorimaea operculella*, belongs to the family Gelechiidae in the order Lepidoptera [18]. *Phthorimaea operculella* is primarily a pest of potatoes but also can be found in other solanaceous crops including tomato, tobacco, pepper and cape gooseberry [19]. It has been reported that *P. operculella* utilizes 60 alternate hosts of cultivated and wild plants and most of them belong to the Solanaceae family [20]. *Phthorimaea operculella* adults lay their eggs in leaves, stems, and tubers. The immature stages mine the leaves, resulting in foliar damage and burrow into tubers. Chemical signals play a crucial role in host selection, as the detection of plant odours could prompt the female to seek out the most suitable host for her offspring [19]. Numerous studies have found a direct correlation between the quantity of captured adults and the abundance of larvae on both the leaves and tubers [21]. The tomato leaf miner *Phthorimaea absoluta* belongs to same family Gelechiidae and order Lepidoptera and is also a significant insect pest that severely damages tomato crops. The pest was initially discovered in South America and quickly spread worldwide [22]. Solanaceous species are the main host plants for *P. absoluta*, with tomato, potato, and *Solanum nigrum* being the most preferable [23], but can also lay eggs and develop on various plants from the Amaranthaceae, Convolvulaceae, Fabaceae, and Malvaceae families [24]. Since the 1950s, *P. absoluta* has become a major pest of tomato crops in South America. *Phthorimaea absoluta* primarily damages host plants in their larval stage. The larvae create a narrow leaf mine by mining the leaf mesophyll when feeding and when populations are at high density, the larvae delve beneath sepals, mining through axillary buds in young stems and/or tomato fruits. When infesting potato plants, *P. absoluta* only feeds on the aboveground parts and does not damage the tubers and this can be a distinguishing characteristic from *P. operculella* [25].

Although high-quality genome data for *P. operculella* and *P. absoluta* have been published [26], our understanding of the chemosensory genes of these two insects remains limited. He et al. sequenced the antennal transcriptome of *P. operculella* and analyzed the function of the sex pheromone receptor PR in *P. operculella* [27]. Li et al. analyzed the types and functions of odorant-binding proteins (OBPs) in *P. operculella*, providing important insights for the study of the chemical ecology of *P. operculella* [28]*.* To further investigate the chemical ecological mechanisms of *P. operculella* and *P. absoluta*, comprehensive identification and analysis of their chemosensory genes are urgently needed and potentially of great significance.

In this study, a total of 93 ORs, 206 GRs, and 50 IRs were identified from the genome of *P. operculella*, while 72 ORs, 122 GRs, and 46 IRs were identified from the genome of *P. absoluta*. The gene structure and phylogenetic characteristics of these genes were analyzed. Transcriptional profiling of chemosensory receptor genes from *P. operculella* at different developmental stages and chemosensory organs was conducted. Through differential expressed genes (DEGs) analysis of male vs. female antennae, we found that 32 ORs, 9 GRs, and 4 IRs were DEGs. This study provides a foundation for further research on the chemosensory gene characteristics of *P. operculella* and *P. absoluta*.

## Results

### Identification of chemosensory receptor genes

#### Candidate odorant receptors

Genome analysis of *P. operculella* led to the identification of 93 OR genes containing one co-receptor PopeORco with 92 odorant receptors named PopeOR1 to PopeOR92. Through the combined analysis of the antennal transcriptome and genome, the ORs of the potato tuber moth were well annotated. The 93 odorant receptor genes we identified. Based on transcriptome information and comparative analysis with homologous receptor genes, 75 PopeORs have predicted full-length sequences, with the possibility of having 3–8 transmembrane helical domains. Using similar methods, we annotated 72 odorant receptor genes from the genome of *P. absoluta* through the homology annotation pipeline. These genes include one PabsORco gene and 71 odorant receptor genes named PabsOR71 ~ PabsOR72 (Additional file 2: Table S2).

**Fig. 1:**
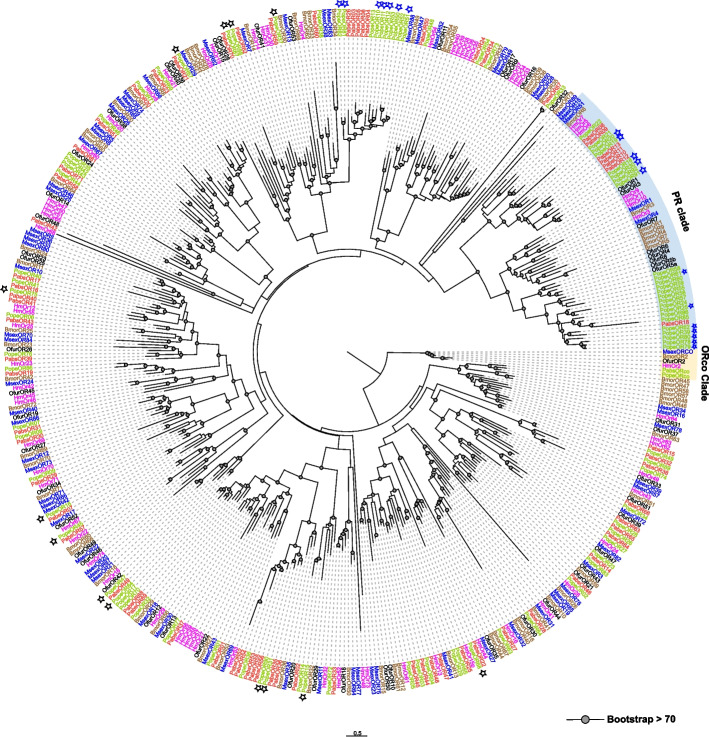
The phylogenetic tree of odorant receptors in Lepidoptera species. The gray dots denote > 70% bootstrap support values. Shaded colors indicate different kinds of ORs: Yellow (ORco clade), and light blue (PR clade). Asterisks represent DEGs in antennae, black asterisks indicate higher expression in females, while blue asterisks indicate differential expression in males

We performed an approximate maximum likelihood phylogenetic analysis of the odorant receptors (ORs) identified in *P. operculella* and *P. absoluta*, along with other publicly available ORs from Lepidopteran species. We observed that 24 PopeORs (PopeOR18 ~ 22, PopeOR24 ~ 25, PopeOR30, PopeOR39, PopeOR55 ~ 62, PopeOR78 ~ 80, and PopeOR85 ~ 86, PopeOR91 ~ 92,) and 7 PabsORs (PabsOR8 ~ 13, PabsOR18) cluster together with the Lepidopteran pheromone receptors (PRs) clade (Table 1, Fig. 1). Additionally, we found that PopeORs and PabsORs have a greater number of OR orthologs. Furthermore, they each exhibit at least one species-specific branch, indicating their distinct evolutionary trajectory (Fig. 1).

**Table 1:** Two *Phthorimaea* pest species chemosensory receptor gene family repertoires

Gene Family	Gene subfamily	*Phthorimaea operculella*	*Phthorimaea absoluta*
Gustatory receptor	CO_2_ receptors	4	4
	Sugar receptors	11	12
	Fructose receptor	12	7
	Bitter receptors	190	105
Odorant receptor	Pheromone receptors	24	7
	Odorant receptor co-receptor	1	1
	General odorant receptor	68	64
Ionotropic receptors	antennal IRs	16	15(1)
	Divergent IRs	25	22
	Lepidoptera-specific IRs	5	5
	Co-Receptor IR genes	4	4

Numbers indicate intact genes and numbers in parentheses indicate pseudogenes

#### Candidate gustatory receptors

A total of 206 gustatory receptors (GRs) were identified from the genome of *P. operculella*, 194 of which have full-length ORFs ranging from 227 to 580 aa in length. Twelve of these 206 GR sequences are partial sequences. Transmembrane domain (TMD) prediction indicates that these full-length PopeGR genes have 4–9 TMDs (Additional file 2: Table S2). A total of 122 GRs were annotated from the genome of *P. absoluta*, and all had intact ORFs encoding protein lengths from 209 to 652 aa which have 4 ~ 9 TMDs. For the nomenclature of *PopeGRs* and *PabsGRs*, we numbered the genes according to their position information on chromosomes. All details about gene information and the structure of gustatory receptor genes are shown in Additional file 2: Table S2.

**Fig. 2:**
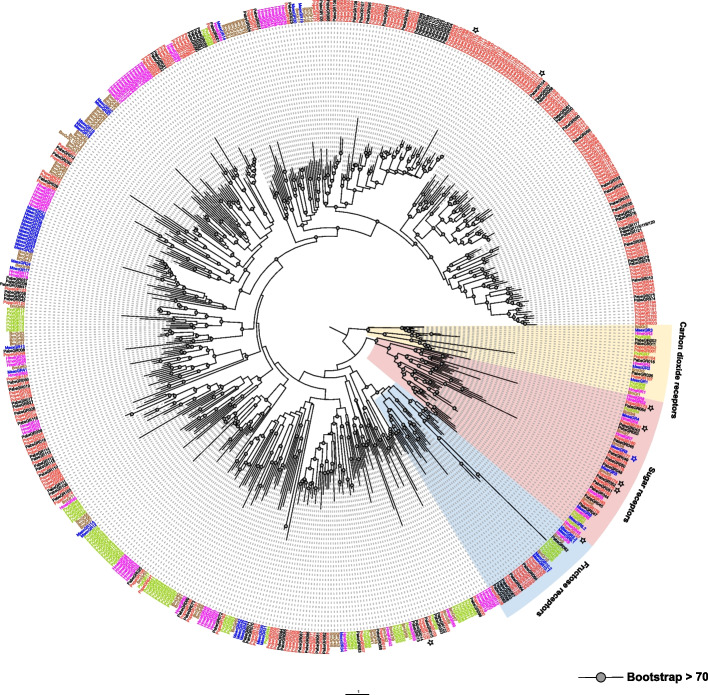
The phylogenetic tree of gustatory receptors in Lepidoptera species. The gray dots denote > 70% bootstrap support values. The shaded colours indicate different types of GRs. Asterisks represent DEGs in antennae, black asterisks indicate higher expression in females, while blue asterisks indicate differential expression in males

A phylogenetic analysis was performed by combining the gustatory receptor sequences from of *P. operculella*, *P. absoluta*, *Bombyx mori*, 69 sequences from *Plutella xylostella*, 45 sequences from *Manduca sexta*, and *Heliconius melpomene.* The ML tree showed that: 11 PopeGRs (PopeGR173 ~ 183) and 12 PabsGRs (PabsGR043 ~ 54) clustered to sugar receptors, 12 PopeGRs (PopeGR125 ~ 126) and 7 PabsGRs (PabsGR62 ~ 68) clustered with the fructose receptor, 4 PopeGRs (PopeGR001, PopeGR042, PopeGR104, and PopeGR107) and 4 PabsGRs (PabsGR002, PabsGR011, PabsGR016, and PabsGR026) clustered with the carbon dioxide receptors, and PopeGRs and PabsGRs clustered with the bitter receptor’s clade (Table 1, Fig. 2).

#### Candidate ionotropic receptors

Overall, we have provided comprehensive descriptions of 50 and 46 IR genes in *P. operculella* and *P. absoluta*, respectively, through our annotations (Additional file 2: Table S2). The IR gene structure of *P. operculella* consisted of 1 to 20 exons, with 26 genes having no introns among the 50 IR genes. Eight genes had a higher number of introns (≥ 14). The average length of these PopeIRs was 659 amino acids (aa), and the length of individual gene models ranged from 389 to 1063 aa (Additional file 2: Table S2). We did not find any evidence of pseudogene or alternative splicing from the *P. operculella* genome and transcriptome data. All the annotated PopeIRs were full-length. The IR gene structure of *P. absoluta* consisted of 1 to 19 exons, with 23 genes having no introns among the 46 IR genes. Six genes had a higher number of introns (≥ 14). The average length of these PabsIRs was 649 aa, and the length of individual gene models ranged from 391 to 1000 aa (Additional file 2: Table S2). From the Pabs genome, we found that PabsIR93a.1 might be a pseudogene, while the remaining 45 PabsIR genes had complete ORFs (Additional file 2: Table S2).

**Fig. 3:**
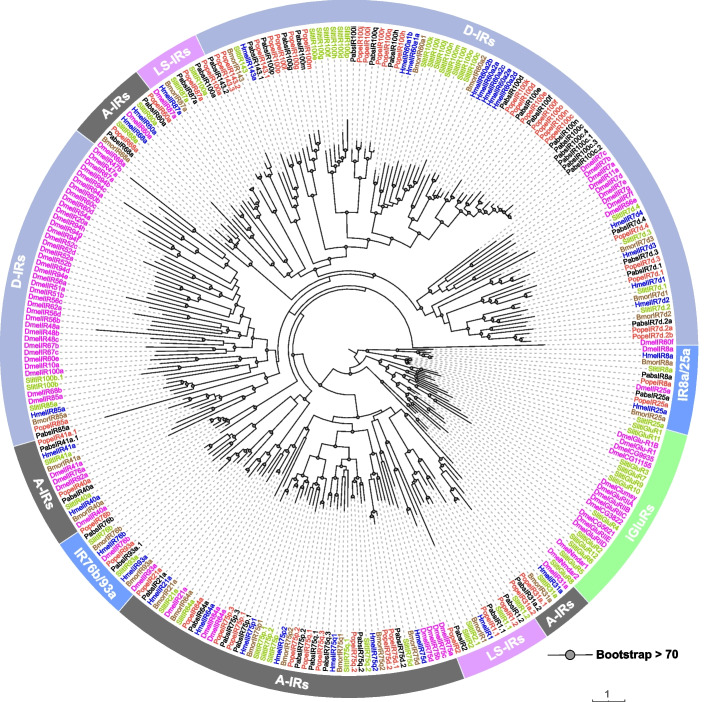
The phylogenetic relationships of ionotropic receptors between Lepidoptera and Diptera species. Gray dots denote > 70% bootstrap support values. Shaded colours indicate different types of IR genes: Divergent IRs (D-IRs clade), Antennal IRs (A-IRs clade), Lepidoptera-specific IRs (LS-IRs clade), Co-Receptor IR genes (IR25a/8a, andIR76b//93a clade)

Phylogenetic analysis and ionotropic receptors from *Drosophila melanogaster* [29] and three Lepidoptera species, including *H. melpomene* [30–32], *B. mori* [30, 31, 33], and *S. litura* [34–36]. The phylogenetic results showed that *P. operculella* and *P. absoluta* have conserved co-receptors IR8a, IR25a, IR76b, and IR93a; In *P. operculella*, 16 candidate PopeIRs were clustered with the “antennal IRs” clade, including PopeIR31a.1/2, PopeIR75p.1/2/3, PopeIR75q.1/2/3, PopeIR75d.1/2, PopeIR21a, PopeIR68a, PopeIR40a, PopeIR60a, PopeIR64a, PopeIR41a, while 25 PopeIRs (PopeIR100c ~ r, PopeIR7d.1/2a/2b/3/4, PopeIR85a, and PopeIR143.1/2/3) belong to the "divergent IRs" family (Fig. 3). The PopeIR1.1/2, PopeIR2, PopeIR100a, and PopeIR87a belong to "Lepidopteran-specific IRs ". In contrast, for *P. absoluta*, 22 PabsIRs were found to belong to the D-IRs branch, including PabsIR100c ~ q, PabsIR143.1/2, PabsIR85a, and PabsIR7d.1 ~ 4. Five PabsIRs (PabsIR1.1, PabsIR1.2, PabsIR100a, PabsIR2, and PabsIR87a) were classified as LS-IRs. Additionally, 15 PabsIRs (PabsIR21a, PabsIR31a.1/2, PabsIR40a, PabsIR41a.1, PabsIR60a, PabsIR64a, PabsIR68a, PabsIR75d, PabsIR75p.1/2/3, PabsIR75q.1/2/3) belong to the antennal IR clade (Fig. 3).

### Chromosomal distribution map of GRs

**Fig. 4:**
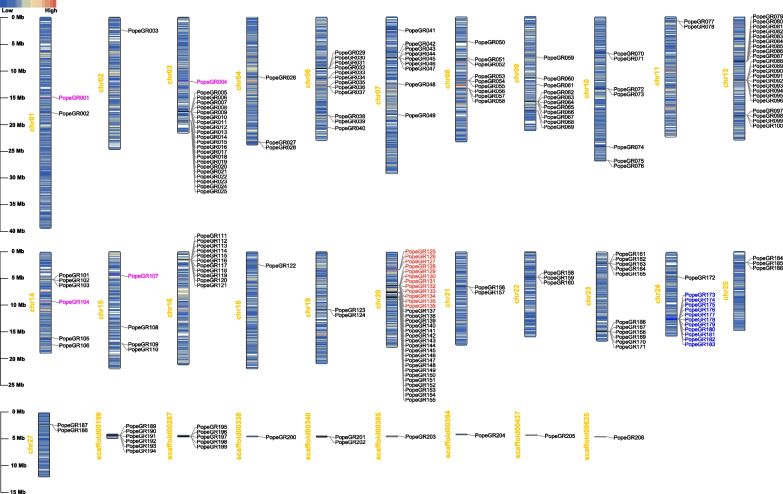
Chromosomal distribution map of *PopeGR* genes. The sugar receptors are marked in blue, fructose receptor is marked in red, and carbon dioxide receptors are marked in magenta

The gustatory receptor (GRs) genes of *P. operculella* were mapped on the chromosomes. The number of chromosomes in *P. operculella* was 29, and the 206 identified GR sequences were distributed on 23 different chromosomes and 8 scaffolds, which include chromosomes 1, 2, 3, 4, 6, 7, 8, 9, 10, 11, 13, 14, 15, 16, 18, 19, 20, 21, 22, 23, 24, 25, 27, Scaffold00199, Scaffold00287, Scaffold00338, Scaffold00340, Scaffold00365, Scaffold00394, Scaffold00437, Scaffold00625 (Fig. 4 and Additional file 2: Table S2).

Overall, the distribution of GR genes in the genome of *P. operculella* is relatively discrete. The number of GR genes on chromosomes 3, 13 and 20 is significantly higher than on other chromosomes, and there is a taste receptor expansion phenomenon on these chromosomes, which is speculated to be produced by large-scale replication of genes. The distribution of sugar receptors in *P. operculella* is relatively concentrated: all are located on Chromosome 24. Fructose receptors are located on chromosome 20. PTM carbon dioxide receptor distribution is relatively scattered, with four carbon dioxide receptors distributed on chromosomes 1, 2, 14 and 15. The bitter taste receptor in the genome of *P. operculella* is also relatively scattered. There is a large-scale amplification phenomenon of bitter receptors on chromosomes 13 and 20, which corresponds to the results of systematic evolution analysis (Figs. 2 and 4).

**Fig. 5:**
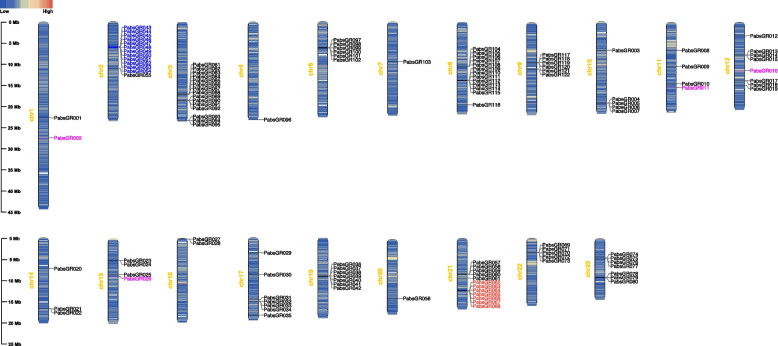
Chromosomal distribution map of *PabsGR* genes. The sugar receptors are marked in blue, fructose receptor is marked in red, and carbon dioxide receptors are marked in magenta

The 122 identified gustatory receptor (GRs) genes of *P. absoluta* were mapped on chromosomes, and it was found that PabsGRs were distributed on the following 20 chromosomes: 1, 2, 3, 4, 6, 7, 8, 9, 10, 11, 12, 14, 15, 16, 17, 19, 20, 21, 22, and 23 (Fig. 5 and Additional file 2: Table S2). Overall, the distribution of GR genes in the genome of *P. absoluta* is also relatively discrete. Among them, the number of GR genes on chromosomes 2, 3, 8 and 21 is significantly higher than on other chromosomes, and there is also an expansion of taste receptors on these chromosomes. The distribution of sugar receptors in *P. absoluta* is also relatively concentrated, but unlike *P. operculella*, all sugar receptors in *P. absoluta* are located on chromosome 2. However, the fructose receptor of *P. absoluta* is also located on chromosome 20. The distribution of carbon dioxide receptors in *P. absoluta* is relatively scattered, with four carbon dioxide receptors distributed on chromosomes 1, 11, 12 and 15 respectively. The bitter taste receptors in *P. absoluta* are also scattered in the genome, with large-scale amplification of bitter receptors on chromosomes 3 and 8 (Fig. 5).

### Analysis of potato tuber moth chemosensory organs transcriptome

We collected and analyzed 11 chemical sensory organs of *P. operculella*, including larval head, adult female antennae, head, legs, and ovipositor, and adult male antennae and genitalia. Through transcriptome sequencing, we obtained a total of 1425.37 million raw reads. After the adapters and low-quality raw reads were filtered out, we obtained 1410.44 million clean reads. In addition, the average Q30 base percentage after filtering was 93.22% (see Additional file 3: Table S3). When the clean reads were aligned to the reference genome of *P. operculella*, the average alignment rate (percentage of aligned reads to clean reads) of the samples was 86% (Additional file 4: Table S4). The raw reads were deposited in the National Center for Biotechnology Information (NCBI) Sequence Read Archive (SRA) database with submission number PRJNA1074269. Gene expression levels of all chemosensory receptor genes based on the transcripts per million (TPM) value are represented in Table S5.

**Fig. 6:**
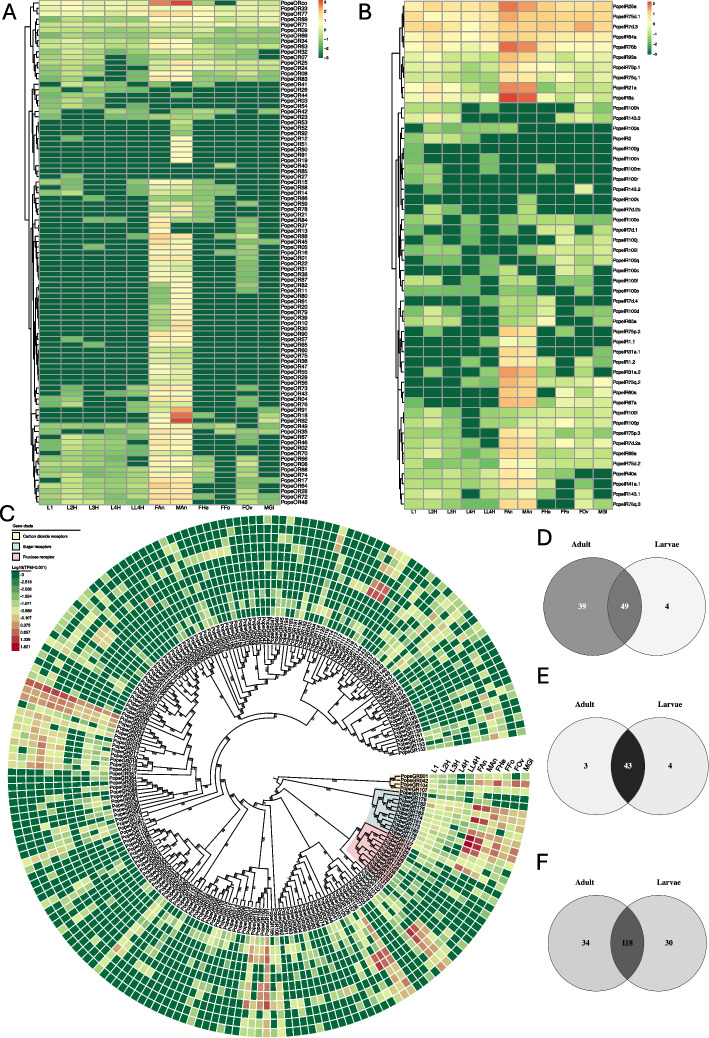
Analysis of expression patterns of chemosensory receptor genes in *Phthorimaea operculella*. All expression values are represented as normalized TPM (Log10 (TPM + 0.001)), and the colour represents the expression level, with red representing high expression and dark green representing low expression. **A** Heat map of expression levels of odorant receptor genes in *P. operculella*; **B**) Heat map of expression levels of ionotropic receptor genes in *P. operculella*; **C**) Phylogenetic tree and heat map of gustatory receptor genes in *P. operculella*, with bootstrap values shown as numbers in the figure, using IQ-Tree, with 1000 bootstraps for tree construction; **D**) Expression information of ORs in adult and larval stages; **E**) Expression information of IRs in adult and larval stages; **F** Expression information of GRs in adult and larval stages: The abbreviations and names of each tissue are as follows: L1: first-instar larvae, L2H: second-instar larvae heads, L3H: third-instar larvae heads, L4H: fourth-instar larvae heads, LL4H: mature larvae heads, FAn: female adult antennae, Man: male adult antennae, FHe: female adult heads, FFo: female adult legs, FOv: female adult ovipositors, MGl: male adult reproductive organs

The expression level analysis of odorant receptor genes showed that almost all ORs were enriched in the antennae. Only one OR (*PopeOR85*) was not detected in any tissue. PopeORco displayed the highest expression level in both male and female antennae, but no expression was detected in the legs. Two ORs (*PopeOR33* and *PopeOR77*) were expressed in all tissues, with PopeOR33 highly expressed in the head of mature larvae, and PopeOR77 highly expressed in the adult antennae (Fig. 6 A and Additional file 5: Table S5). The results of OR expression analysis also showed that 53 ORs were detected in the head of larvae, while 88 ORs were detected in the chemosensory tissues of adults. There were 49 ORs expressed throughout the entire larval and adult stages, 4 ORs (*PopeOR3*, *PopeOR27*, *PopeOR54*, *PopeOR85*) expressed only during the larval period, and 39 ORs expressed only during the adult period (Fig. 6 D and Additional file 5: Table S5). Through DEGs analysis, it was found that 12 *PRs* (*PopeOR18*-*20*, *PopeOR25*, *PopeOR30*, *PopeOR39*, *PopeOR61*-*62*, *PopeOR78-80*, *PopeOR91*) had higher expression levels in the antennae of males than females. The remaining 12 *PRs* had no significant difference in expression levels between the two sexes. Thirteen ORs (*PopeOR15*, *PopeOR16*, *PopeOR31*, *PopeOR37*, *PopeOR45*, *PopeOR47*, *PopeOR67-68*, *PopeOR75*, *PopeOR84*, *PopeOR87*-*88*, *PopeOR90*) had significantly higher expression levels in female antennae than males (Fig. 6A and Additional file 6: Table S6).

The expression analysis of ionotropic receptor genes showed that the main antennal IRs were enriched in the antennae, while the expression levels of divergent IRs, which are specific to Lepidoptera, did not show obvious tissue specificity. Interestingly, we found that some antennal IRs, including the *PopeIR25a/76b* clade, PopeIR75d clade, PopeIR64a clade, and divergent IRs (PopeIR7d.3), had high expression levels in all tissues (Fig. 6B and Additional file 5: Table S5). The results of IR expression analysis also showed that 47 IRs were detected in the head of larvae, while 46 IRs were detected in the chemosensory tissues of adults. All IRs were expressed throughout the entire larval and adult stages, while 4 IRs expressed only during the larval period and 3 IRs expressed only during the adult period (Fig. 6E and Additional file 5: Table S5). Six A-IRs (PopeIR75p.2, PopeIR31a.1, PopeIR31a.2, PopeIR75q.2, PopeIR60a) and three LS-IRs (PopeIR1.1, PopeIR1.2, PopeIR87a) were mainly expressed in adult tissues. Two LS-IRs (PopeIR100a PopeIR2) and seven D-IRs (PopeIR100g, PopeIR100n, PopeIR100m, PopeIR100r, PopeIR143.2, PopeIR100k, PopeIR7d.2b) were mainly expressed in larval tissues (Fig. 6B). The analysis of DEGs in male and female antennae showed that four A-IRs (PopeIR40a, PopeIR75d.2, PopeIR75p.1, PopeIR75p.2) had significantly higher expression levels in female antennae than in male antennae (Table S6).

The results of GRs expression analysis also showed that 148 GRs were detected in the head of larvae, while 152 GRs were detected in the chemosensory tissues of adults. There were 182 GRs expressed throughout the entire larval and adult stages, 30 GRs expressed only during the larval period, and 34 GRs expressed only during the adult period (Fig. 6C and F). Among these GRs, four carbon dioxide receptors in potato tuber moth were expressed in adult and larval tissues, of which PopeGR107 was only detected in the antennae of female adults and the heads of L1 ~ 4 larvae, while PopeGR042 was mainly expressed in adult tissues than in larvae. All sugar receptors had higher expression levels in adult tissues than in larvae. The sugar receptor PopeGR182 had higher expression levels in the heads of 1st instar larvae, 3rd instar larvae and female adults. The fructose receptor PopeGR127 was expressed in various tissues, with higher expression levels in the antennae of the adult and female heads (Fig. 6 C). The expression analysis of these bitter gustatory receptors revealed that four bitter taste receptors (PopeGR022, PopeGR024, PopeGE156, PopeGR161) had high expression levels in all tissues (Fig. 6C). From Fig. 6C, the constructing an evolutionary tree of 206 PopeGRs and labelling their expression information shows these bitter receptors cluster on one branch of the evolutionary tree and have similar expression patterns, such as the bitter taste receptor PopeGR015 ~ PopeGR019 branch all being highly expressed in adult tissues.

The analysis of DEGs in male and female antennae revealed distinct patterns. Female adults exhibited significantly higher expression levels of four sugar receptors PopeGRs (PopeGR173–175, PopeGR181) and fructose receptor PopeGR127 in their antennae compared to male adults. On the other hand, the sugar receptor PopeGR177 had significantly higher expression levels in the antennae of male adults compared to female adults. Additionally, among the bitter taste receptors, PopeGR027, PopeGR102 and PopeGR111 exhibited significantly higher expression levels in the antennae of female adults compared to male adults (Fig. 6C and Table S6).

## Discussion

The development of sequencing technology has made it possible to study the size and evolutionary relationships of chemosensory gene families of different species [1, 37], providing a good reference to annotate and study the evolution of chemosensory genes in two *Phthorimaea* pests. In this study, we conducted genome-based identification of chemosensory receptor genes in *P. operculella* and *P. absoluta*. Our objective was to investigate the numbers, types, and evolutionary relationships of chemosensory receptor-related genes in two closely related *Phthorimaea* pests with similar habits and evolutionary relationships. By manually annotating the genomes of these two insect species, we identified 349 (93 *PopeORs*, 206 *PopeGRs*, 50 *PopeIRs*) and 240 (72 *PabsORs*, 122 *PabsGRs*, 46 *PabsIRs*) candidate chemosensory receptor genes from the genomes of *P. operculella* and *P. absoluta*, respectively. These data allow exploration of the molecular mechanisms underlying their mutual adaptation and competition. Furthermore, we hope that this study will lay the foundation for further research in their chemical ecology and the development of environmentally friendly pest control techniques based on insect olfaction.

### A complete annotation of odorant receptors in two Phthorimaea pests provides insights for inter-specific competition and host selection

**Fig. 7:**
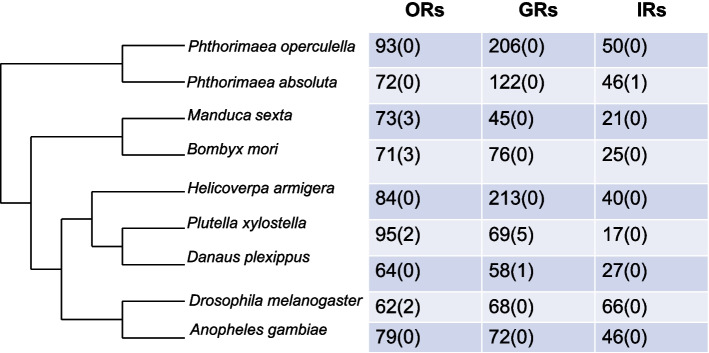
Insect chemosensory receptors gene family statistics. The numbers indicate genes and numbers in parentheses indicate pseudogenes. ORs indicate olfactory receptors, GR indicates gustatory receptors, and IRs indicate ionotropic receptors

Researchers have found that insect ORs are a highly variable family of receptor genes by comparing the sequences of various insect ORs. A total of 79 different ORs were discovered in the genome of *Anopheles gambiae* [38, 39]. By comparing the OR families of *D. melanogaster* and *A. gambiae*, it was found that although both belong to Diptera, their OR sequences differ greatly, and there is gene expansion to varying degrees in each OR subfamily (Fig. 7) [39]. Some insects with complex environments, such as the red wood ant *Pogonomyrmex barbatus*, have up to 345 ORs. However, an exception was found in *Tribolium castaneum*, a storage pest that can complete its entire life cycle within a limited food range. Despite this, it has 245 ORs, suggesting that its environment may be relatively complex, and adults rely on a well-developed olfactory system to locate food resources [40, 41]. These findings reflect that the number and sequence of ORs are generally adapted to the ecological needs of each species. Research on the number and phylogenetic analysis of insect ORs can provide valuable information on the evolution and functional differentiation of insect ORs [42]. From the number of ORs in *P. operculella* and *P. absoluta*, it can be seen that *P. operculella* (93 ORs) has a larger number of odorant receptor genes compared to *B. mori* (71 ORs) [30, 33], *H. melpomene* (70 ORs) [30], and *P. absoluta* (72 ORs), and is comparable to the number of genes in *P. xylostella* (95 *ORs*) [43]. Evolutionary analysis of these ORs revealed that the pheromone receptor branches in these lepidopteran insects are relatively conserved and clustered together, which is consistent with previous studies [44]. Among them, the PR branch of *P. operculella* showed species-specific expansion with a total of 24 branches, and the bootstrap values for these branches were all above 70%. Similar results were reported in *P. xylostella*, where Engsontia et al*.* identified many species-specific branch expansions in the PR. However, due to the lack of functional evidence, these branches were temporarily classified as putative PR branches [43]. For consideration for classification as candidate pheromone receptors, at least one of them should exhibit male-specific expression associated with hairy sensilla, according to the idea proposed by Koenig et al. [45]. Therefore, in this study, these ORs are also temporarily classified as PR branches, and further functional studies are needed to analyze the expression and functional characteristics of these putative PRs. However, in *P. absoluta*, we only identified seven PRs and uncovered evidence of a close evolutionary relationship between the two species in terms of PRs. In addition, the large number of expanded branches in PopePRs suggests that there are also large differences in the recognition of sex pheromone components between the two species. For example, Chang et al. studied the PRs of two *Helicoverpa* species and found that functional studies of the PRs of two closely related moths, *Helicoverpa armigera* and *H. assulta*, revealed that homologous PRs have maintained functional consistency during the evolutionary process, although most of the similar receptors have maintained functional consistency, however, there is also the possibility that species divergence events may have caused certain PR direct homologs to produce functional consistency [46].

Through expression profile analysis of these PopePRs, we found that not all the predicted PRs exhibited male-biased expression in the male moths of the potato tuber moth, with only 12 PRs showing male-specific or highly expressed patterns. This indicates that these PRs may be involved in male perception of female sex pheromones. Other PRs did not show significant expression preferences. The study by Wan et al. on the codling moth demonstrated that the sex pheromone receptor branch of *CpomOR3a* and *CpomOR3b* exhibited female-biased expression, and these PRs were not only involved in sex pheromone perception but also the perception of host plant volatiles [47]. Therefore, it can be speculated that the function of these PopePRs is that they do not exhibit clear male-biased expression preferences. In addition to participating in the perception of sex pheromones, these PRs may also be involved in the perception of other chemical cues from sources such as host plant volatiles. In addition, in *B. mori*, studies have found that BmorOR19, BmorOR45, and BmorOR47 are receptors that show female-biased expression. They respond to aromatic volatile compounds such as linalool, benzoic acid, and benzaldehyde. It has been suggested that these receptors may be involved in regulating oviposition behaviour or detecting male sex pheromones [48]. According to DEGs analysis, we found 13 PopeORs that showed significantly higher expression levels in female antennae. It is speculated that these *PopeORs* may be involved in the oviposition behaviour of females. *PopeOR77* is highly expressed throughout the growth and development stages. Interestingly, PopeOR77 and BmorOR66 cluster together on the same branch. BmorOR56 has a similar expression pattern in the silkworm and is highly sensitive to cis-jasmone and is involved in the chemo-attraction response to cis-jasmone [33]. This suggests that PopeOR77 in *P. absoluta* is likely to have similar biological functions as BmorOR77 in the silkworm.

### The extensive expansion and expression pattern analysis of bitter taste receptors provide new insights into the mechanisms of host adaptation in oligophagous insect species

Insect GRs can detect non-volatile compounds in the environment through contact chemosensation of amino acids, sugars, bitter substances, and some plant secondary metabolites. Each species has a unique number of GRs. Engsontia et al. suggested that the expansion of Gr genes is mainly due to extensive gene duplications and relatively few gene losses [43]. In addition, it has been shown that the total number of GRs may be closely related to species behaviour and their ecological adaptation. For example, in model species such as fruit flies and mosquitoes (*D. melanogaster* and *Anopheles gambiae*), 60 and 76 *Gr* genes have been identified, respectively. In a human parasitic louse *Pediculus humanus,* only 6 GR genes were found, indicating a correlation between the low number of GRs and the simple environment needed for this insect to survive [49]. *Helicoverpa armigera* is currently reported to have the highest number of gustatory receptors, with 213 GRs identified (Fig. 7) [50]. In some lepidopteran species with narrower host range, such as *B. mori*, 76 GRs have been identified [51], while *D. plexippus* has 64 GRs [32, 52], *Heliconius melpomene* has 70 GRs [30], and *P. xylostella* has 69 GRs [43]. Among these four species, the silkworm feeds exclusively on mulberry leaves [48, 53], *D. plexippus* feeds only on plants of the Asclepiadaceae family [52], the *Heliconius melpomene* feeds exclusively on *Passiflora oerstedii* or *P. menispermifolia* [30], and *P. xylostella* only feeds on plants of the Brassicaceae family [54]. Therefore, the expansion of 213 GRs in the cotton bollworm may be related to its feeding habits, as the extensive expansion of GR branches may enhance its ability to perceive a wide range of host plant metabolites, facilitating its survival on various host species [50]. In our study, we found an interesting phenomenon: the number of GRs identified in the specialist herbivore *P. operculella* is much higher than those in the generalist herbivore *P. absoluta*. Phylogenetic analysis of their GRs revealed no significant differences in the number of carbon dioxide receptors, sugar receptors, and two types of gustatory receptors. Furthermore, through expression studies, it has been found that the differential expression of taste receptor genes on the antennae of the potato tuber moth is mainly related to sugar receptors and fructose receptors. It is speculated that this may be associated with the adult insects' need for increased sugar perception to supplement the energy required for mating, oviposition, and flight, especially in females. Interestingly, we observed that more than half of the female moths showed a preference for the expression of genes enriched in sugar receptors. In our analysis of fructose receptor expression in the potato tuber moth, we found that the *PopeGR127* fructose receptor gene is expressed to varying degrees in chemosensory tissues at different developmental stages. Other putative fructose receptors are predominantly expressed in the heads of larvae, suggesting their possible involvement in regulating feeding-related behaviours. Further research is needed to investigate the expression and functional characteristics of these sugar receptors in the gut and other tissues. In addition, there is substantial evidence of expansion in the fructose receptor branch of the potato tuber moth, a conserved class of GRs found in almost all insects. Studies of fruit flies and cotton bollworms have shown that fructose receptors may function as internal nutrient sensors, playing important roles in regulating feeding behaviour [55, 56].

Notably, *P. operculella* showed a significant expansion in the branches of fructose and bitter receptors. *Phthorimaea operculella* is a specialized herbivorous lepidopteran insect that exclusively feeds on plants of the Solanaceae family, such as potatoes and eggplants. It is speculated that the specific expansion of bitter taste receptor branches in *P. operculella* helps it better perceive the various glycoalkaloids commonly present in solanaceous plants [57], allowing it to select hosts that are beneficial for its survival. The potato tuber moth may adapt to solanaceous hosts by using a large expansion of bitter receptors to selectively feed on suitable hosts or parts of plants. Analysis of DEGs (differentially expressed genes) between the sexes of the potato tuber moth revealed that three bitter taste receptor genes were highly expressed in the female antennae. We speculate that this may be related to the female moth's perception and oviposition behaviour.

### Identification of the complete set of ionotropic receptors in two *Phthorimaea* pests provides crucial insights into their physiological characteristics and management strategies

A total of 50 and 46 IRs were identified from *P. operculella* and *P. absoluta*. This is a higher number compared to other lepidopteran species *B. mori*, and *D. plexippus* (Fig. 7) [31]. Ionotropic receptor genes were first identified in fruit flies and are divided into A-IRs and D-IRs [13] based on their expression characteristics and evolutionary relationships. Studies on Lepidoptera have found that insects in the Family Noctuidae have a unique IR branch called LS-IRs [58]. Subsequent studies on ionotropic receptors in Lepidoptera have found that some LS-IRs are not exclusive to the noctuids, such as IR1 [45]. Similarly, we also identified the IR1 branch in *P. operculella* and *P. absoluta*. Like the silkworm, we did not identify the 100b branch of the LS-IR in *P. operculella* and *P. absoluta*, but Zhu et al. identified the IR100b branch in *S. litura*. By comparing the types of D-IR receptors in *P. operculella* and *P. absoluta*, we found that the number of ionotropic receptor genes in both species was the same, and no species-specific IR receptor branches were found. The differences between the two species are mainly due to the fact that the PabsIR100c branch has four additional gene copies (PabsIR100c.1 − 4) located close to each other on the chromosome, suggesting similar physiological functions. However, we did not observe an expansion of the PopeIR100c branch in *P. operculella*. We found three gene copies on the PopeIR143 branch, while only one gene copy was found on the PabsIR143 branch. The same phenomenon was found in the IR75d and IR7d branches. Analysis of exon and intron structures of these two species’ IR genes revealed that the gene structures of LS-IRs (except for IR1.1 and IR1.2) and D-IRs are relatively simpler than those of conserved A-IRs, showing fewer introns or single exon structures. For example, D-IR subfamilies (except for IR85a) lack introns, while A-IR subfamilies usually have multiple introns, like fruit fly IRs [59]. Interestingly, we found one intron in two D-IRs of PabsIR (PabsIR100c.1 and PabsIR143.2). Unlike dipterans, the IR85a branch in both species' D-IRs is a single exon gene. In A-IRs, we also found that the IR68 branch is a single exon gene similar to that in *Spodoptera litura*; however, the structure of IR68a is more complex and different from that in *Spodoptera litura* [35].

Based on the expression profile analysis of ionotropic receptors (IRs) in the chemosensory tissues of *P. operculella* during developmental stages, we found some interesting expression patterns. For example, IR25a, IR76b, IR8a, IR64a and IR75d were enriched in the antennae of different developmental stages. Previous studies have shown that these receptors may be involved in the olfactory and gustatory perception of acids by various insects [60–62]. These acids may come from the products of microbial fermentation of sugars [63]. Therefore, the conserved expression characteristics of these antennal IRs suggest that they play an important role in the perception of acids throughout the entire development of *P. operculella*. PopeIR7d.4, which belongs to Divergent-IR, also shows a similar expression pattern. However, there is little research on the function of Divergent-IRs in Lepidoptera, and further study is needed to determine the function of this gene. The expression pattern analysis of other D-IRs also suggests that these IRs are not enriched in adult antennae, and some are only detected in the heads of larvae, suggesting that they may be related to feeding and olfaction functions. For example, D-IRs involved in the taste perception of some fatty acids have been found in fruit flies [64]. Further research is needed to fully understand the function of D-IRs. Different Pope LS-IRs show various expression patterns. We particularly note that PopeIR100a is expressed not only in the heads of larvae but also in the female antennae of adults, suggesting that it may be related to olfaction function. However, there is limited research on its function and further functional verification is needed. The diverse expression profiles of the potato tuber moth indicate different expression patterns, which may reflect different ecological adaptation strategies and behavioural characteristics. Current research indicates that ionotropic receptor genes in insects have multiple sensory functions, including olfaction, taste, temperature, and humidity perception [65]. Specifically, IRs play a role in the temperature and humidity perception of pests, for example, IR25a and IR93a are expressed in thermosensory neurons and humidity-sensing neurons in the antennae, participating in temperature and humidity perception [15]. Furthermore, some IRs, such as IR21a, are essential for temperature-based host-seeking behavior in mosquitoes [66], while other IRs are involved in the perception of specific chemicals in the environment, such as amino acids and organic acids [67–69]. Some organic acids provide insects with rich information. For example, the accumulation of large amounts of acids indicates severe food resource corruption, and at the same time, the accumulation of acids is disadvantageous to the survival of insects [70, 71]. The perception of acids helps insects assess the suitability of food resources and help them to avoid eating toxic and harmful areas. Our study shows that the homology of the ionotropic receptor gene sequences of these two pests was close, and the specific expression patterns of some ionotropic receptor genes in different tissues and developmental stages reveal that these ionotropic receptors play important roles in olfaction, gustation, and the perception of temperature and humidity. These genes can serve as potential targets for interference with the host plant recognition of these two pests, providing sufficient data support and molecular targets for further research on the development of pest management strategies.

## Conclusion

In this study, we investigated the chemosensory receptor-related genes of *P. operculella* and *P. absoluta* in terms of their similar habits and evolutionary relationships. In addition, the odorant receptor and ionotropic receptor genes of both insect species were identified, and the close homology of these chemosensory receptor genes revealed similarities in the functions of receptors between the two species. These two types of receptors are mainly involved in olfactory perception, which also suggests the possibility of intense interspecific competition in olfactory behaviour between the two species. Our research demonstrated that the potato tuber moth has more chemosensory receptor genes (349 genes) compared to the tomato leaf miner (240). We discovered extremely large expansions of bitter receptor genes in the *P. operculella* compared to the *P. absoluta*, which may be related to the broad adaptation to solanaceous hosts of *P. operculella*. These research findings lay the foundation for further studies on the chemical ecology of these pests and the development of environmentally friendly pest control technologies based on insect chemosensation.

## Methods

### Sample collection and sequencing

*Phthorimaea operculella* were collected from potato fields in QuJing, Yunnan Province, China, in 2014. In the laboratory, the rearing conditions in the climate controlled chamber were as follows: temperature, 26 ± 1°C; relative humidity, 60 ± 10%; photoperiod, 12 h light: 12 h dark. The larvae were reared using potatoes and placed together with the adults in nylon cages. The amount of head tissues used for each larval stage were as follows: L1 (whole head larvae, approximately 50 individuals), L2 larvae (approximately 90 heads dissected), L3 larvae (approximately 80 heads dissected), L4 larvae and mature larvae (approximately 50 heads dissected). The antennae (80 pairs of each sex), heads (40 females), legs (40 females), male genitalia (40 males), and female ovipositors (40 females) were separately excised from 2 − 3-day-old adults and immediately frozen in liquid nitrogen and stored at − 70 ℃ until use.

The total RNA of the tissues above was separately extracted using TRIzol reagent (Invitrogen, Carlsbad, CA, USA) following the manufacturer’s instructions. RNA integrity was determined with an Agilent Bioanalyzer 2100 system (Agilent Technologies Inc., CA, USA). RNA concentration and purity were measured by a Nanodrop ND2000 spectrophotometer (NanoDrop Technologies Inc., Wilmington, DE). One microgram of total RNA per sample was used for cDNA library construction. A cDNA library was prepared using the Optimal Dual-mode mRNA Library Prep Kit (BGI, Shenzhen, China) according to the manufacturer's instructions. The library was then sequenced on the BGI MGISEQ-2000 platform (BGI, Shenzhen, China), and paired-end (150 bp) reads were generated.

### Identification chemosensory genes

Comparative genomic analysis was conducted between *P. operculella* and *P. absoluta* [18, 72]. Chemosensory gene sequences were collected from various lepidopteran species with published genome and transcriptome data, including *Bombyx mori* [30, 31, 33], *Plutella xylostella* [73], *Chilo suppressalis* [74, 75], *Ostrinia furnacalis* [74, 76], *Helicoverpa armigera* [50], *Helicoverpa zea* [50], *Galleria mellonella* [74, 77], *Eogystia hippophaecolus* [78], *Spodoptera exigua* [74, 79], *Peridroma saucia* [80], *Carposina sasakii* [81], *Mythimna separata* [82–84], *Manduca sexta* [45, 85, 86], *Loxostege sticticalis* [87], *Danaus plexippus* [31, 32, 52], *Heliconius melpomene* [30–32], and *Spodoptera litura* [34–36] (Additional file 1: Table S1). Next, protein sequences were obtained from the National Center for Biotechnology Information (https://www.ncbi.nlm.nih.gov) based on published data and manually verified to construct a reliable reference dataset.

Subsequently, a local TBLASTN (v2.14.0) analysis was performed to search candidate chemosensory genes in the genomes of *P. operculella* and *P. absoluta*, with an E-value cutoff of e^−5^ [88]. Based on the Blast search results, the positional information of different chemosensory genes on chromosomes or scaffolds was manually determined. The genome sequence of these candidate receptor genes was extracted using a Python script, and the coding sequences of chemosensory genes from the genomes of the two species were identified using an online server Genewise (https://www.ebi.ac.uk/Tools/psa/genewise/). Furthermore, after obtaining RNA-seq data of chemosensory tissues in the potato tuber moth, we aligned the clean reads to the reference genome of the potato tuber moth using the HISAT software (v2.10) [89]. Then, we assembled the aligned reads using StingTie (v1.0.4) to reconstruct transcript information. Based on the transcript information, we corrected the sequences of chemosensory genes annotated from the genome, resulting in nearly complete genes including exon–intron boundaries and their location in the potato tuber month. Furthermore, the conserved domains of these candidate genes were confirmed using the InterPro online server (https://www.ebi.ac.uk/interpro/search/sequence/) [90].

### Sequence and phylogenetic analysis

We used the ORF prediction component of the TBtools software (v2.034) to predict the open reading frames (ORFs) of chemosensory genes in species *P. operculella* and *P. absoluta* [91]. The transmembrane helical domains (TMDs) of chemosensory genes were predicted using the online server DeepTMHMM (https://dtu.biolib.com/DeepTMHMM). TBtools was used to visualize the chromosomal positions of gustatory receptor genes in *P. operculella* and *P. absoluta* [91].

All chemosensory gene sequence alignments were performed by the MAFFT software (v7.490). Then, we used ModelFinder to find the best amino acid substitution model for phylogenetic analysis. The phylogenetic trees for chemosensory genes were constructed using IQ-Tree software v.2.0.7) based on the best amino acid substitution model, with 1000 bootstraps. We used the OR dataset, including sequences from *Bombyx mori* [33], *Ostrinia furnacalis* [76], *Manduca sexta* [45], and *Heliconius melpomene* [30]. The GR phylogenetic trees included sequences from *Bombyx mori* [51], *Plutella xylostella* [43], *Manduca sexta*, and 73 from *Heliconius melpomene* [32]. The IR dataset included sequences from *Drosophila melanogaster* [29], *Heliconius melpomene* [31], *Bombyx mori* [31], and *Spodoptera litura* [35]. The sequences resource of each species was collected from genome or transcriptome data (see Additional file 1: Table S1). The ML tree was visualized using iTOL online tools (v. 5) [92].

### *P. operculella*’s chemosensory genes expression analysis

We conducted a study on the developmental stages and tissue expression profiles of chemosensory genes in the potato tuber moth. The raw reads were processed using SOAPnuke software (v1.6.5) to eliminate unknown (poly N) or low-quality and adaptor sequences, resulting in clean data. Quality metrics, including Q20, Q30, and GC-content levels, were calculated for the clean data. All subsequent analyses were performed using high-quality clean data. Subsequently, the clean reads were aligned to the reference genome using STAR (2.7.10b) [93]. Furthermore, we employed RSEM to calculate the gene expression levels in each tissue [94]. Finally, the expression levels of the chemosensory genes were visualized using the pHeatmap package (v1.0.12) in R (v4.1.2). For the differentially expressed gene (DEG) analysis, we used the R package DEseq2 to analyze the number of differentially expressed chemosensory genes between female and male antennae [95]. We set | Log2 (FoldChange) |> 1, FDR < 0.05 as the threshold for screening differentially expressed genes.

### Supplementary Information

Additional file 1: Table S1. The information of reference chemosensory gene set used in gene annotation from other species.

Additional file 2: Table S2. The detail of identification of chemosensory receptor genes.

Additional file 3: Table S3. Quality statistics of Clean Reads.

Additional file 4: Table S4. Summary of genome mapping information.

Additional file 5: Table S5 Table. Chemosensory gene expression data.

Additional file 6: Table S6. DEGs information between FAn Vs MAn. Set FoldChang > 2, FDR < 0.05 as threads to filter DEGs.

## Data Availability

No datasets were generated or analysed during the current study.

## Abbreviations

ORs: Odorant receptors
GRs: Gustatory receptors
IRs: Ionotropic receptors
GPCR: G protein-coupled receptor
CO2: Carbon dioxide
OSNs: Olfactory sensory neurons
GRNs: Gustatory receptor neurons
OBPs: Odorant-binding proteins
DEGs: Differential expressed genes
TMD: Transmembrane domain
PR: Pheromone receptor
D-IRs: Divergent IRs
LS-IRs: Lepidopteran-specific IRs
A-IRs: Antennal IRs
