# Supplementary material for: Genomic identification and evolutionary analysis of chemosensory receptor gene families in two Phthorimaea pest species: insights into chemical ecology and host adaptation
Source: BMC Genomics. 2024 May 18;25:493. doi: 10.1186/s12864-024-10428-6 (PMC11102633; doi:10.1186/s12864-024-10428-6)
Supplement: Supplementary file 1 — Additional file 1: Table S1. The information of reference chemosensory gene set used in gene annotation from other species. [file 12864_2024_10428_MOESM1_ESM.docx]

**Table S1 The information of reference chemosensory genes from lepidoptera and Dipteran species.**

| Species name | OR | GR | IR | Reference type |
| --- | --- | --- | --- | --- |
| *Bombyx mori* | 71[1, 2] | 76[3] | 25[4] | Genome |
| *Plutella xylostella* | 95[5] | 69[5] | 16[6] | Transcriptome & Genome |
| *Chilo suppressalis* | 47 | — | 36[7] | Transcriptome & Genome [8] |
| *Ostrinia furnacalis* | 54 | 5 | 39[7] | Genome [9, 10] |
| *Helicoverpa armigera* | 84 | 213 | 40 | Genome[11] |
| *Helicoverpa zea* | 82 | 166 | 40 | Genome [11] |
| *Galleria mellonella* | 43 | —— | 45[7] | Transcriptome & Genome [12] |
| *Eogystia hippophaecolus* | 63 | 13 | 12 | Transcriptome [13] |
| S*podoptera exigua* | 63 | 38 | 44[7] | Genome [14] |
| *Peridroma saucia* | 63 | 10 | 24 | Transcriptome [15] |
| *Carposina sasakii* | 52 | 11 | 8 | Transcriptome [16] |
| *Mythimna separata* | 67[17] | 16[18] | 19[17] | Transcriptome |
| *Manduca sexta* | 73[19] | 45[19] | 21[19] | Genome |
| *Loxostege sticticalis* | 54 | 13 | 18 | Transcriptome [20] |
| Danaus plexippus | 64[21, 22] | 58[21, 22] | 27[4] | Genome |
| *Heliconius melpomene* | 70[2] | 73[22] | 31[4] | Genome |
| *Spodoptera litura* | 73[23] | 237[23] | 45[24] | Transcriptome & Genome |
| *Drosophila melanogaster* | 62[25, 26] | 68[27] | 66[28] | Genome |

Note: “—” indicates that were not found sequence from National Center for Biotechnology Information (<https://www.ncbi.nlm.nih.gov>) and articles.

# Reference

1. Tanaka K, Uda Y, Ono Y, Nakagawa T, Suwa M, Yamaoka R, et al. Highly selective tuning of a silkworm olfactory receptor to a key mulberry leaf volatile. Curr Biol. 2009;19(11):881-90. Epub 20090507. doi: 10.1016/j.cub.2009.04.035. PMID: 19427209.

2. Heliconius Genome C. Butterfly genome reveals promiscuous exchange of mimicry adaptations among species. Nature. 2012;487(7405):94-8. doi: 10.1038/nature11041. PMID: 22722851; PubMed Central PMCID: PMC3398145.

3. Guo H, Cheng T, Chen Z, Jiang L, Guo Y, Liu J, et al. Expression map of a complete set of gustatory receptor genes in chemosensory organs of Bombyx mori. Insect Biochem Mol Biol. 2017;82:74-82. Epub 20170207. doi: 10.1016/j.ibmb.2017.02.001. PMID: 28185941.

4. van Schooten B, Jiggins CD, Briscoe AD, Papa R. Genome-wide analysis of ionotropic receptors provides insight into their evolution in Heliconius butterflies. BMC Genomics. 2016;17:254. Epub 20160322. doi: 10.1186/s12864-016-2572-y. PMID: 27004525; PubMed Central PMCID: PMC4804616.

5. Engsontia P, Sangket U, Chotigeat W, Satasook C. Molecular evolution of the odorant and gustatory receptor genes in lepidopteran insects: implications for their adaptation and speciation. J Mol Evol. 2014;79(1-2):21-39. Epub 20140720. doi: 10.1007/s00239-014-9633-0. PMID: 25038840.

6. Yang S, Cao D, Wang G, Liu Y. Identification of Genes Involved in Chemoreception in Plutella xyllostella by Antennal Transcriptome Analysis. Sci Rep. 2017;7(1):11941. Epub 20170920. doi: 10.1038/s41598-017-11646-7. PMID: 28931846; PubMed Central PMCID: PMC5607341.

7. Yin NN, Nuo SM, Xiao HY, Zhao YJ, Zhu JY, Liu NY. The ionotropic receptor gene family in Lepidoptera and Trichoptera: Annotation, evolutionary and functional perspectives. Genomics. 2021;113(1 Pt 2):601-12. Epub 20200928. doi: 10.1016/j.ygeno.2020.09.056. PMID: 33002624.

8. Cao D, Liu Y, Wei J, Liao X, Walker WB, Li J, et al. Identification of candidate olfactory genes in Chilo suppressalis by antennal transcriptome analysis. Int J Biol Sci. 2014;10(8):846-60. Epub 20140726. doi: 10.7150/ijbs.9297. PMID: 25076861; PubMed Central PMCID: PMC4115196.

9. Yang B, Ozaki K, Ishikawa Y, Matsuo T. Identification of candidate odorant receptors in Asian corn borer Ostrinia furnacalis. PLoS One. 2015;10(3):e0121261. Epub 20150324. doi: 10.1371/journal.pone.0121261. PMID: 25803580; PubMed Central PMCID: PMC4372370.

10. Yang B, Ozaki K, Ishikawa Y, Matsuo T. Sexually biased expression of odorant-binding proteins and chemosensory proteins in Asian corn borer Ostrinia furnacalis (Lepidoptera: Crambidae). Applied Entomology and Zoology. 2016;51(3):373-83. doi: 10.1007/s13355-016-0409-4. PMID: WOS:000380066300005.

11. Pearce SL, Clarke DF, East PD, Elfekih S, Gordon KHJ, Jermiin LS, et al. Genomic innovations, transcriptional plasticity and gene loss underlying the evolution and divergence of two highly polyphagous and invasive Helicoverpa pest species. BMC Biol. 2017;15(1):63. Epub 20170731. doi: 10.1186/s12915-017-0402-6. PMID: 28756777; PubMed Central PMCID: PMC5535293.

12. Jiang XC, Liu S, Jiang XY, Wang ZW, Xiao JJ, Gao Q, et al. Identification of Olfactory Genes From the Greater Wax Moth by Antennal Transcriptome Analysis. Front Physiol. 2021;12:663040. Epub 20210519. doi: 10.3389/fphys.2021.663040. PMID: 34093226; PubMed Central PMCID: PMC8172125.

13. Hu P, Tao J, Cui M, Gao C, Lu P, Luo Y. Antennal transcriptome analysis and expression profiles of odorant binding proteins in Eogystia hippophaecolus (Lepidoptera: Cossidae). BMC Genomics. 2016;17:651. Epub 20160818. doi: 10.1186/s12864-016-3008-4. PMID: 27538507; PubMed Central PMCID: PMC4989532.

14. Llopis-Gimenez A, Carrasco-Oltra T, Jacquin-Joly E, Herrero S, Crava CM. Coupling Transcriptomics and Behaviour to Unveil the Olfactory System of Spodoptera exigua Larvae. J Chem Ecol. 2020;46(11-12):1017-31. Epub 20201105. doi: 10.1007/s10886-020-01224-z. PMID: 33150456.

15. Sun YL, Dong JF, Gu N, Wang SL. Identification of Candidate Chemosensory Receptors in the Antennae of the Variegated Cutworm, Peridroma saucia Hubner, Based on a Transcriptome Analysis. Front Physiol. 2020;11:39. Epub 2020/02/23. doi: 10.3389/fphys.2020.00039. PMID: 32082194; PubMed Central PMCID: PMC7005060.

16. Tian Z, Sun L, Li Y, Quan L, Zhang H, Yan W, et al. Antennal transcriptome analysis of the chemosensory gene families in Carposina sasakii (Lepidoptera: Carposinidae). BMC Genomics. 2018;19(1):544. Epub 20180720. doi: 10.1186/s12864-018-4900-x. PMID: 30029592; PubMed Central PMCID: PMC6053724.

17. Tang R, Jiang NJ, Ning C, Li GC, Huang LQ, Wang CZ. The olfactory reception of acetic acid and ionotropic receptors in the Oriental armyworm, Mythimna separata Walker. Insect Biochem Mol Biol. 2020;118:103312. Epub 20200102. doi: 10.1016/j.ibmb.2019.103312. PMID: 31904488.

18. Du L, Zhao X, Liang X, Gao X, Liu Y, Wang G. Identification of candidate chemosensory genes in Mythimna separata by transcriptomic analysis. BMC Genomics. 2018;19(1):518. Epub 20180704. doi: 10.1186/s12864-018-4898-0. PMID: 29973137; PubMed Central PMCID: PMC6030794.

19. Koenig C, Hirsh A, Bucks S, Klinner C, Vogel H, Shukla A, et al. A reference gene set for chemosensory receptor genes of Manduca sexta. Insect Biochem Mol Biol. 2015;66:51-63. Epub 20150911. doi: 10.1016/j.ibmb.2015.09.007. PMID: 26365739.

20. Wei HS, Li KB, Zhang S, Cao YZ, Yin J. Identification of candidate chemosensory genes by transcriptome analysis in Loxostege sticticalis Linnaeus. PLoS One. 2017;12(4):e0174036. Epub 20170419. doi: 10.1371/journal.pone.0174036. PMID: 28423037; PubMed Central PMCID: PMC5396883.

21. Zhan S, Merlin C, Boore JL, Reppert SM. The monarch butterfly genome yields insights into long-distance migration. Cell. 2011;147(5):1171-85. doi: 10.1016/j.cell.2011.09.052. PMID: 22118469; PubMed Central PMCID: PMC3225893.

22. Briscoe AD, Macias-Munoz A, Kozak KM, Walters JR, Yuan F, Jamie GA, et al. Female behaviour drives expression and evolution of gustatory receptors in butterflies. PLoS Genet. 2013;9(7):e1003620. Epub 20130711. doi: 10.1371/journal.pgen.1003620. PMID: 23950722; PubMed Central PMCID: PMC3732137.

23. Cheng T, Wu J, Wu Y, Chilukuri RV, Huang L, Yamamoto K, et al. Genomic adaptation to polyphagy and insecticides in a major East Asian noctuid pest. Nat Ecol Evol. 2017;1(11):1747-56. Epub 20170925. doi: 10.1038/s41559-017-0314-4. PMID: 28963452.

24. Zhu JY, Xu ZW, Zhang XM, Liu NY. Genome-based identification and analysis of ionotropic receptors in Spodoptera litura. Naturwissenschaften. 2018;105(5-6):38. Epub 20180522. doi: 10.1007/s00114-018-1563-z. PMID: 29789962.

25. Clyne PJ, Warr CG, Freeman MR, Lessing D, Kim J, Carlson JR. A Novel Family of Divergent Seven-Transmembrane Proteins: Candidate Odorant Receptors in Drosophila. Neuron. 1999;22(2):327-38. doi: <https://doi.org/10.1016/S0896-6273(00)81093-4>.

26. Gao Q, Chess A. Identification of Candidate Drosophila Olfactory Receptors from Genomic DNA Sequence. Genomics. 1999;60(1):31-9. doi: <https://doi.org/10.1006/geno.1999.5894>.

27. Robertson HM, Warr CG, Carlson JR. Molecular evolution of the insect chemoreceptor gene superfamily in Drosophila melanogaster. Proc Natl Acad Sci U S A. 2003;100 Suppl 2(Suppl 2):14537-42. Epub 20031107. doi: 10.1073/pnas.2335847100. PMID: 14608037; PubMed Central PMCID: PMC304115.

28. Croset V, Rytz R, Cummins SF, Budd A, Brawand D, Kaessmann H, et al. Ancient protostome origin of chemosensory ionotropic glutamate receptors and the evolution of insect taste and olfaction. PLoS Genet. 2010;6(8):e1001064. Epub 20100819. doi: 10.1371/journal.pgen.1001064. PMID: 20808886; PubMed Central PMCID: PMC2924276.
